# An analyser for the continuous determination of acrolein in the atmosphere

**DOI:** 10.1155/S1463924682000303

**Published:** 1982

**Authors:** Janet F. Reddish

**Affiliations:** 1 Analytical Section Research Department Imperial Chemical Industries (Petrochemicals and Plastics Division) PLC Hertfordshire Welwyn Garden City AL7 1HD UK; 2 107 High Street Codicote Hertfordshire SG4 8UB UK


Journal of Automatic Chemistry, Volume 4, Number 3 (July–September 1982), pages 116–121

### An analyser for the continuous determination of acrolein in the atmosphere

**Janet F. Reddish**

*Analytical Section, Research Department, Imperial Chemical Industries (Petrochemicals and Plastics Division) PLC, Welwyn Garden City, Hertfordshire AL7 1HD, UK**

### Introduction

For some years, interest has been increasing in the nature and amount of volatile products and fumes produced during the processing, thermal degradation and combustion of plastics. Of particular concern has been the toxic and irritant effects of aldehydes including formaldehyde, acrolein and crotonaldehyde, which are produced mainly from polyolefines and polyesters.

The author's initial work was carried out principally by a high performance liquid chromatographic method in which the sample atmosphere was drawn through two ice-cooled absorbers in series, containing 2,4-dinitrophenylhydrazine in dilute hydrochloric acid, at a rate of 1 l/min. The resultant 2,4-dinitrophenylhydrazone derivatives were extracted into methylene chloride and the solution so produced chromatographed on a Spherisorb S5W (100 × 5 mm) column using two mixed solvent eluents. Derivatives of formaldehyde, acrolein, acetaldehyde, propionaldehyde crotonaldehyde, butyraldehyde, and valeraldehyde could be quantitatively resolved and this procedure was ideal for short/medium-term sampling. The method, now updated to the reverse phase conditions used by Kuwater *et al.* [1] does, however, require that the analysis be completed in the laboratory, and does not give 'real time' information continuously over long periods during process or degradation/combustion experiments.

It was therefore necessary to develop an analyser specifically for acrolein, which would be capable of a lower limit of determination of 0·01 ppm in air. The requirement was for continuous operation for reasonably long periods (up to 8 h) and for results to be available within a delay time of less than 20 min. The colorimetric method due to Cohen and Altshuller [2] based on the formation of a coloured complex between acrolein and 4-hexylresorcinol under specified conditions was chosen as the chemistry for the analyser. This method offers a high degree of specificity; the only species known to react similarly, but to a much lower extent, is erotonaldehyde (approximately 200:1 acrolein to crotonaldehyde at 605 nm for similar concentrations). The development involved the design of a fast-flow atmosphere sampling and reaction system and assembly of the necessary flow-through cell, measurement and recording instrumentation.

### Construction of the analyser

The analyser is constructed from six functional units assembled as shown in figure 1.

### *Regulated gas-sampling units*

The sample flow rate needed to obtain a result within the analysis range of the equipment varies between 100 and 1000 ml/min., as indicated in table 1. For accurate results, the sampling rate has to be constant and this is achieved by means of a suitable flow-stat valve and calibrated flow meter used in conjunction with a suitable pump.

### *Gas scrubber/reaction vessel*

To achieve a detection limit below that of the TLV for acrolein (0·1 ppm) a large volume of sample atmosphere has to be scrubbed by a small volume of reagent. The scrubber, illustrated in figure 2(*a*), was designed for sampling rates of between 250 and 1000 ml/min. and comprises a glass vessel with a 10-turn inclined spiral in conjunction with a bubble-breaker jet incorporated into the reagent delivery tube. This design proved an effective compromise between exhaustive scrubbing and minimal damping of changes in acrolein concentration. A smaller version using a six-turn spiral was made for sampling rates below 250 ml/in (see figure 2[*b*]).

### *Multi-channel peristaltic pump*

A peristaltic pump capable of flow rates on at least two channels of between 0·5 and 5·0 ml/min. is required. In the work described in this paper, an in-house design pump was used but any good quality peristaltic pump should prove adequate. An important point to note is that it is essential, with the corrosive reagent system recommended, that only acid-resistant fluorinated rubber pump tubing and transfer tubing should be used. Polytetrafluorethylene tubing is also suitable for solvent transfer purposes.

### *Reaction coil at 60°C*

The reaction takes place in a 13 m × 1·6 mm ID glass reaction coil maintained at 60°C in a thermostatted bath. More than one coil can, of course, be accommodated in the bath to provide sufficient delay at reagent flow-rates faster than 2 ml/min.

### *Optical flow-through cell*

A flow-through cell of 4 cm path-length using fibre optics to facilitate measurement of the transmission/absorbance was constructed, as shown in figure 3, from a block of black polytetrafluorethylene (PTFE). Glass was found to be unsuitable for the cell as the incident light was transmitted to the second light pipe via the glass walls and effectively halved the sensitivity of the cell. PTFE proved to be a better medium in other respects also; the size of the optical glasses was independent of the bore size of the cell, whereas the bore shape of a

* *Present address: 167 High Street, Codicote, Hertfordshire SG4 8UB, UK.*

 0142 0453/82/0403 0116 $05·00 © 1982 Taylor & Francis Ltd

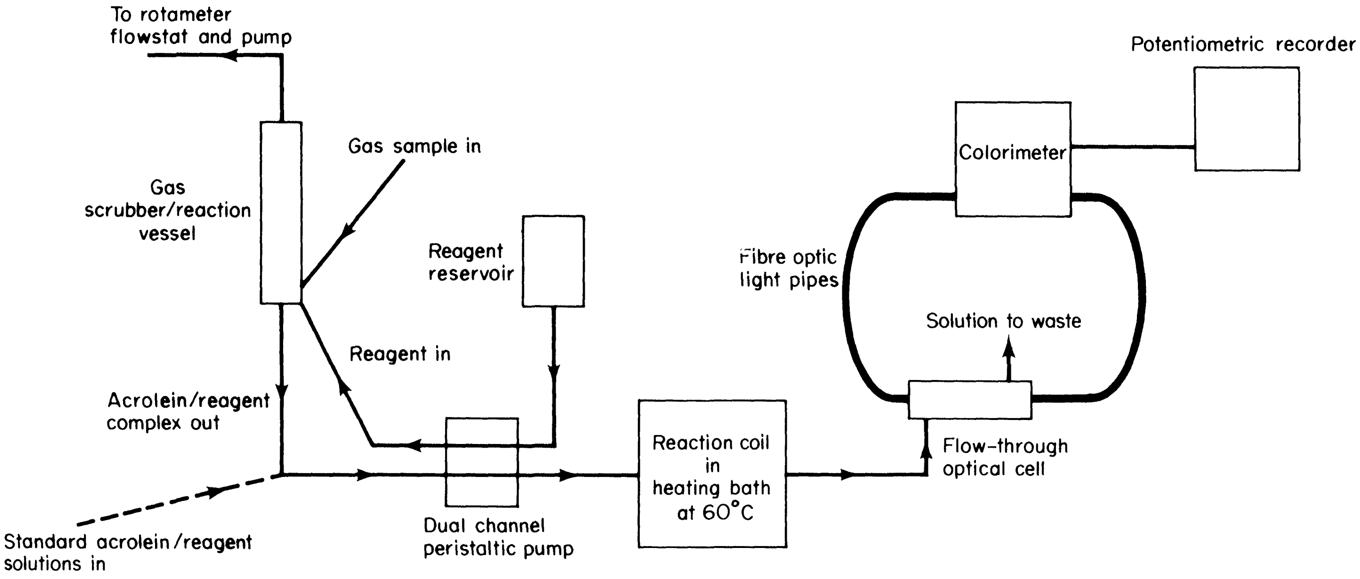

*Figure 1. Schematic layout of continuous acrolein analyser.*

glass cell with integral glasses was determined by the minimum workable size of the glasses. Thus the cell ends had to bell out and so tended to provide bubble traps. With the plastic cell an effective seal could be made between the glass window and the cell body using Viton 'O' rings and the cell bore could therefore relate directly to the cross-sectional area of the light pipes.

### *Colorimeter*

A colorimeter capable of accepting fibre optic light transmission is required. In the assembly described, the fibre optics were obtained from Chemlab and were used in conjunction with a Brinkman dipping probe colorimeter capable of displaying a digital read-out, which could be calibrated directly in concentration units. The absorbance wavelength required for measuring the coloured complex is 605 nm; an interference filter of 600 nm with a 20 nm band pass was used with the colorimeter.

### *Chart recorder*

A permanent record of the concentration/absorbance profile of acrolein was needed and with the colorimeter used, a recorder capable of accepting a 100 mV output was used. The recorder trace obtained from the Brinkman colorimeter is logarithmically related to concentration but this has the advantage that small changes in low levels of acrolein are effectively visually enhanced.

Particulars of the commercially available components used are listed below.

*Apparatus*

| | |
|---|---|
| Vacuum pump: | 1/4 HP oil-filled vacuum pump or Austen Dymax I mini pump. |
| Flowmeter: | GAP 60–600 ml/min. or 100–1200 ml/min., 0–1 or 0–51 air/min. (Flowmeters and Flostat are available from G. A. Platon Ltd, Basingstoke, Hampshire, UK.) |
| Heating bath: | Chemlab High Temperature Bath with 2 × 13 m coils 1·6 mm ID Chemlab Part No. CHB 40. |
| Colorimeter: | Brinkman PC/600D available through Chemlab Ltd, Hornchurch, Essex, UK. |
| Recorder: | single-channel variable mV range pen recorder. |
| Transfer tubing: | thick-wall PTFE tubing about 1 mm ID; 2–3 mm OD available from HETP, Jarman Crest, Jarman Road, Sutton, Macclesfield, Cheshire, UK. |
| Peristaltic pump tubes: | acid-resistant pump tubes available from Gradko International Ltd, Winchester, Hampshire, UK. Flow 1·5 ml/min. 0·074 in. ID green; flow 3 ml/min. 0·110 in. ID purple/white. |
| Connecting nipples: | plastic connectors N5-N9, available from Gradko International Ltd, Winchester, Hampshire, UK. |
| Sampling line: | PVC, PTFE or acid-resistant tubing, as short in length as possible to avoid losses due to condensation. Silicone tubing is not suitable as any contact with the reagent will cause it to disintegrate. |
| Microlitre syringes: | 1, 10, 50, 250 μl Hamilton, SGE or TERUMO glass syringes. |

### Chemicals and reagents required

### *Safety notes*

Trichloroacetic acid/hexylresorcinol reagent: trichloroacetic acid is a very hygroscopic solid forming a most corrosive solution. The final reagent mixture contains about 40–50% w/v trichloroacetic acid, as well as minor amounts of 4-hexylresorcinol and mercuric chloride both of which are toxic. When preparing the various individual solutions for the reagent mixture, handling the reagent, fresh or spent, or dealing with any spillages, however minor, it is very important to wear gloves and eye protection.

Acrolein is a highly toxic aldehyde, TLV 0·1 ppm. It can also polymerize accompanied by an explosion even when stored in a refrigerator. Acrolein should be stored in small volume phials in a freezer. It should always be handled in a fume cupboard, particularly when purifying by vacuum distillation or when preparing calibration standards in ethanol.

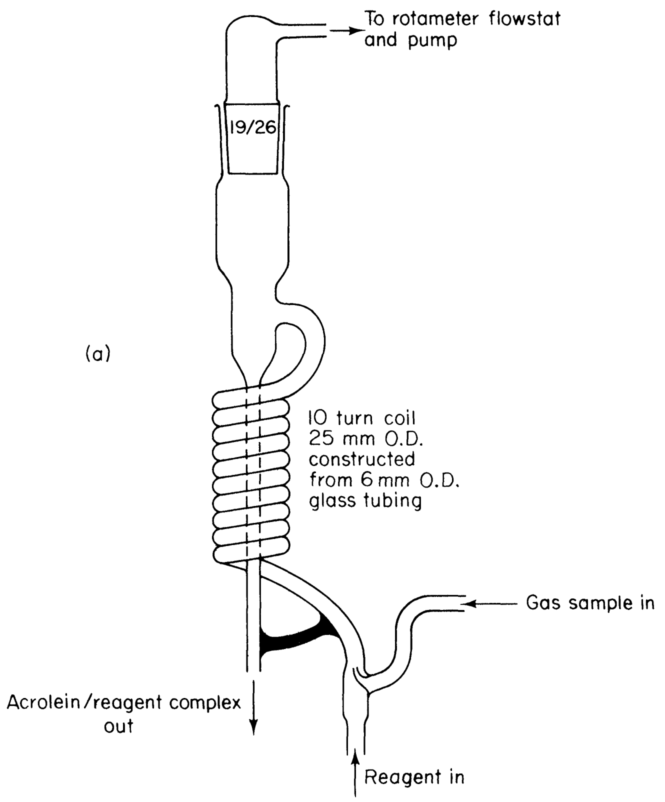

*Figure 2(a). Scrubber for sampling 250–1000 ml/min.*

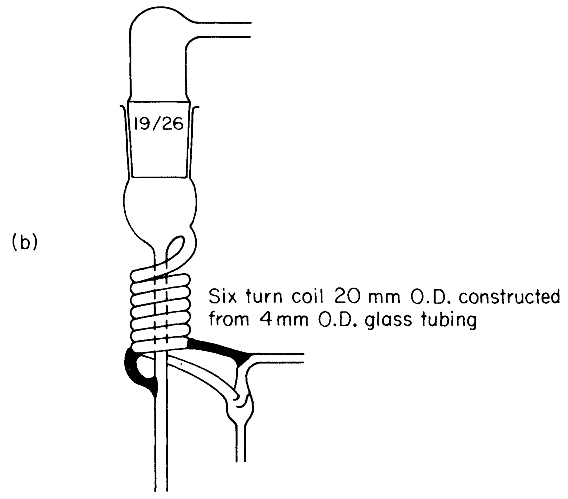

*Figure 2(b). Scrubber for sampling <250 ml/min.*

### *Preparation*

All reagents used should be of analytical reagent quality.

#### *Stock solutions*

Saturated trichloroacetic acid (TCA acid): dissolve 500 gm TCA acid in 50 ml distilled water with warming on a water bath.

96% v/v ethyl alcohol (EtOH): dilute absolute alcohol to 96% v/v with distilled water.

3% w/v mercuric chloride: dissolve 3 gm $HgCl_2$ in 96% EtOH and dilute to 100 ml.

4-hexyl-resorcinol: dissolve 5 g of 4-hexylresorcinol in 5 ml of 96% EtOH.

Reagent mixture: prepared fresh daily for use. Mix 125 ml saturated TCA acid and 5 ml 3% w/v mercuric chloride in EtOH with either 2·5 ml 4-hexylresorcinol solution or 1·25 gm of solid and dilute to 250 ml with 96% ethyl alcohol.

Acrolein.

Acrolein calibration standard: dilute 2·5 μl pure acrolein to 25 ml with 96% EtOH in a grade 'A' volumetric flask.

### Details of analyser operation

The operational range of the analyser is 0·01–17 ppm, which is achievable within the modes of operation detailed in table 1.

*Table 1. Modes of operation of the analyser.*

| Acrolein level ppm | Air sampling rate ml/min | Reagent flow rate ml/min. |
|---|---|---|
| <1 | 500–1000 | 1·5 |
| 1–4 | 250–500 | 1·5 |
| 2–10 | 100–250 | 1·5 |
| 10–17 | 100–250 | 3·0 |

The analyser is normally calibrated using acrolein standards in reagent solution, the preparation of which are described under the section on 'Calibration of the analyser'. Calibration can also be effected using an acrolein/air atmosphere, details of this method are also found in the 'Calibration' section.

The reagent flow rate must be measured at an early stage in order to calculate the acrolein in air levels represented by the solution standards (see table 2). The procedure is as follows.

The apparatus is assembled as indicated in figure 1, but without connecting the gas sampling cell. The heating bath is then switched on and the temperature control set to 60°C. The chart recorder is switched on without engaging the chart drive, next the colorimeter is switched on for use in accordance with the manufacturer's instructions.

A length of transfer tubing is connected to the scavenge peristaltic tube, as indicated in figure 1, and immersed into a reservoir of fresh, cool reagent. The pump is started and pumping of reagent is allowed to continue for at least enough time for the complete passage of liquid through the system: this is the residence time. The pumping is continued until the percentage transmission of the colorimeter has been adjusted and stabilized at 100%. The volume of effluent over a specific

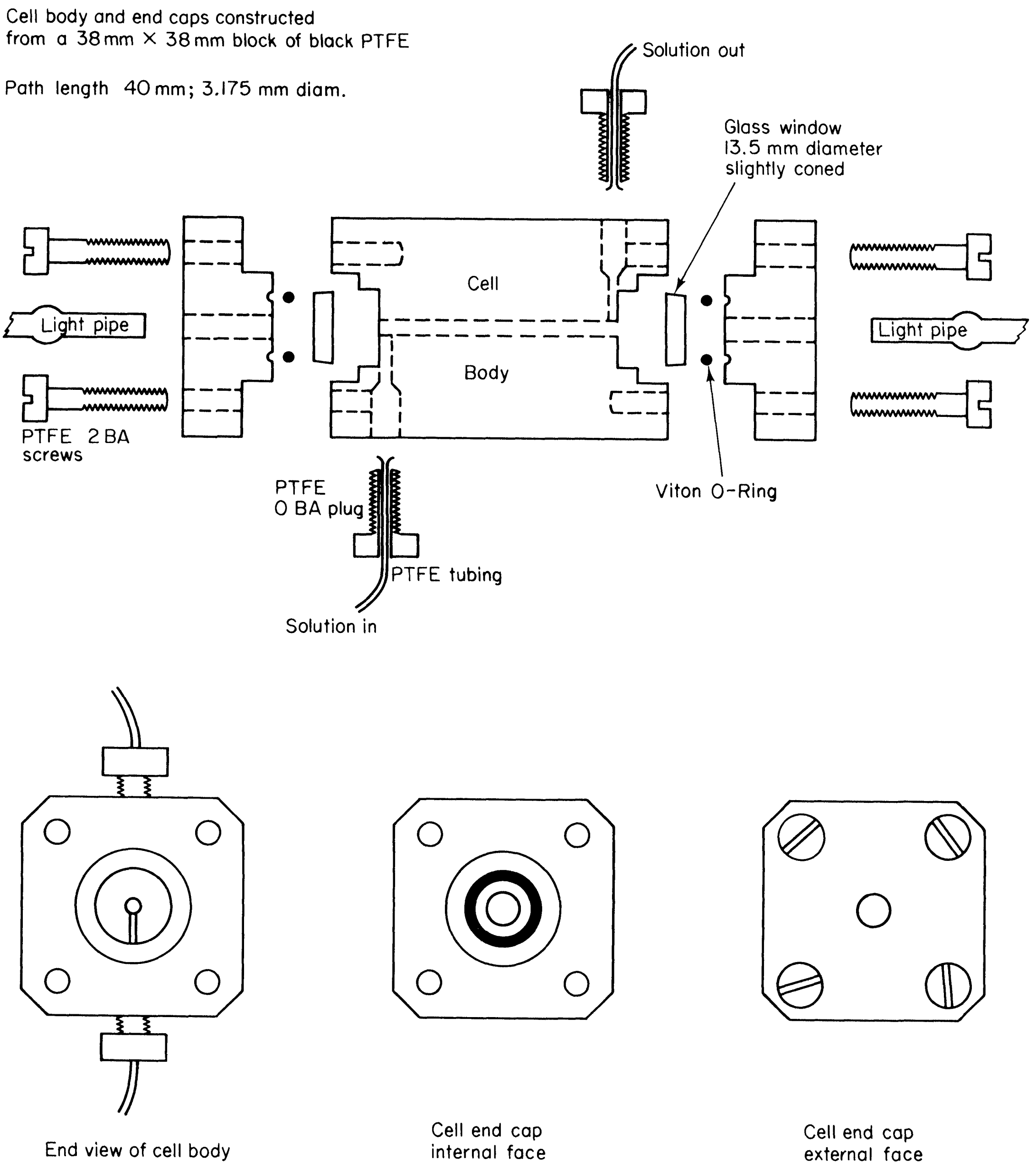

*Figure 3. Optical flow-through cell showing constructional detail.*

time period, for example 10 min., is collected and measured and the pump rate is calculated as:

$$\text{Pump rate} = \frac{\text{Volume of effluent}}{\text{Collection time}} (\text{ml/min.}).$$

The colorimeter is then switched to measure absorbance and adjusted to zero with the appropriate control; the chart drive is turned on. The tube from the reagent has to be quickly transfered to standard 1 and the solution allowed to pump through the system for 10 min. before transferring to standard 2 and so on. The absorbance remains at zero until standard 1 reaches the optical cell. If a bubble has accidentally been introduced into the system, its passage through the optical cell will cause a sharp increase in absorbance and it may take two or more minutes to restabilize.

As the first developed standard passes through the cell the absorbance rises and steadies. At the steady state, the colorimeter is set to indicate the concentration. The read-out is adjusted until it displays the actual concentration of the standard. Every 10 min. the subsequent standards will pass through the optical cell and, in each case, when the absorbance has stabilized, the value is noted and checked against the true concentration value.

After the standards have passed through the system the peristaltic pump is switched off. The delivery and scavenge lines are connected to the appropriate gas sampling cell, the delivery peristaltic pump tube is tensioned, the gas sample pump is turned on and the sampling flow rate set with the Flo-stat controller. The peristaltic pump is then turned on and it is necessary to watch carefully that the gas sampling cell primes satisfactorily. The way that the level of liquid at the top of the down tube is maintained is watched. It may be necessary to constrict either the delivery or scavenge tube by means of a screw clip, or other device, in order to keep the level constant and at a minimum head.

At this point the analyser is ready to monitor a sample atmosphere.

*Table 2. Relationship of atmosphere levels of acrolein to calibration standards.*

| Gas flow : reagent flow | | 1000 ml : 1·5 ml | | 250 ml : 1·5 ml | | 100 ml : 3 ml | | 4 cm Abs. |
|---|---|---|---|---|---|---|---|---|
| Standard | μg Acrolein per 20 ml | mg/M³ | ppm | mg/M³ | ppm | mg/M³ | ppm | 600 nm |
| 1 | 0·3 | 0·022 | 0·010 | | | | | 0·02 |
| 2 | 0·83 | 0·062 | 0·026 | 0·25 | 0·11 | 1·25 | 0·55 | 0·06 |
| 3 | 1·66 | 0·124 | 0·053 | 0·50 | 0·21 | 2·50 | 1·05 | 0·12 |
| 4 | 4·15 | 0·31 | 0·133 | 1·24 | 0·54 | 6·20 | 2·7 | 0·30 |
| 5 | 8·30 | 0·62 | 0·266 | 2·48 | 1·07 | 12·4 | 5·4 | 0·60 |
| 6 | 16·60 | 1·24 | 0·53 | 5·00 | 2·1 | 25·0 | 10·7 | 1·20 |
| 7 | 20·75 | 1·55 | 0·66 | 6·20 | 2·7 | 31·0 | 13·4 | 1·50 |
| 8 | 26·30 | | | | | 39·3 | 17·0 | 1·9 |

### Calibration of the analyser

### *Preparation of solution calibration standards*

10, 20, 50, 100, 200, 250 μl aliquots of the standard acrolein stock solution are added to 20 ml volumes of reagent mixture, using a 50 or 100 μl syringe. These calibration standards are referred to as standards 1–6. Table 2 is a guide to the equivalent acrolein in air concentrations, according to the gas sampling and reagent flow conditions, when solution calibration standards are used. Values are calculated on the following basis:

Acrolein, molecular weight 56, has a specific gravity of 0·83. The stock solution contains 2·5 μl acrolein/ 25 ml ≡ 2·085 mg/ml = 0·0834 μg/μl.

At a reagent flow rate of 1·5 ml/min. a 20 ml calibration standard represents:

$$\frac{20}{1{\cdot}5} = 13{\cdot}33 \text{ min. sampling time.}$$

At 1 l/min. gas sample flow rate, the total gas sample equals 13·33 l. For standard 1 containing 10 μl stock solution, or 0·83 μg acrolein, the relative acrolein in air concentration would be 0·83/13·33 = 0·062 μg/l or mg/M³.

Assuming acrolein to be an ideal gas, its molecular weight in grams occupies 24 l at room temperature.

$$\therefore 0{\cdot}062 \text{ mg/M}^3 \equiv \frac{0{\cdot}062 \times 24}{56} \text{ ppm}$$

$$= 0{\cdot}027 \text{ ppm acrolein.}$$

For pump rates different from 1·5 ml or 3·0 ml, as appropriate the acrolein levels may be recalculated using the expression

$$\frac{\text{ppm acrolein} \times \text{pump rate ml}}{1{\cdot}5 \text{ or } 3{\cdot}0}.$$

It is not recommended that the pump rate be above 2·0 ml/min. for a single passage through the bath as the dwell time will then be less than 13 min. and the optimum development time will not have elapsed.

### *Calibration with a standard acrolein atmosphere*

An alternative and complementary method of calibration, which includes using the gas sampling system, is to prepare a standard acrolein atmosphere in air at a level calculated to produce a reaction colour of absorbance between 0·3 and 0·6 (50–25% transmission), for example an atmosphere of 2 ppm with a gas sampling rate of 100 ml/min.

### *Preparation of acrolein atmosphere*

Using a dynamic atmosphere preparation system an ambient air flow of 3 l/min. is introduced into a 30 L Tedlar air sample bag for 10 min. During this time 0·2 μl or pure acrolein is injected using a 1 μl glass syringe, through the inlet septum tee'd into the air inlet arm. Complete volatilization of the acrolein is ensured by warming the injection T with a hair-dryer. The air flow is diverted and the bag closed at precisely 10 min.

$$0{\cdot}2\,\mu\text{l acrolein} = 0{\cdot}166 \text{ mg}$$

$$166\,\mu\text{g acrolein/30 L air} = 5{\cdot}5 \text{ mg/M}^3$$

$$= 2{\cdot}3 \text{ ppm.}$$

With reference to the calibration chart, this level of acrolein when sampled at 100 ml/min. using a reagent flow of 1·5 ml/min. yields an absorbance of 0·53.

### Shutting-down the analyser

On completion of the measurements the whole system is flushed with 96% ethanol for at least 20 min. and then allowed to pump dry. The peristaltic pump, the gas sampling pump, the heating bath and the colorimeter are switched off and the tension on the peristaltic tubing released.

If the apparatus is likely to be left for some weeks without use, the optical cell should be dismantled, the end glasses washed and cleaned, and the gas sampling cell washed and packed in a protective box.

### Efficiency of gas absorption and precision and accuracy of the analyser

The efficiency of the scrubbers, whilst sampling for acrolein in air up to 1 l/min., have been shown to be equivalent to two impingers in line which indicates an efficiency of about 95% [1].

Provided that grade 'A' glass-measuring equipment (BS 1583, 1792), precision syringes (±2% accuracy) and calibrated flowmeters (nominal accuracy 1·25% but allowing ±5% in practice) are used in the preparation of solution or gaseous calibration standards, the analyser will yield results accurate to better than ±20%; greater accuracy should be achieved using a gas standard calibration, as the efficiency of the scrubber is not then a factor in the calculation. In practice, using the solution calibration method, 90% accuracy has been achieved when measured against determinations made using 2,4 dinitrophenyl hydrazine derivatization followed by HPLC examination.

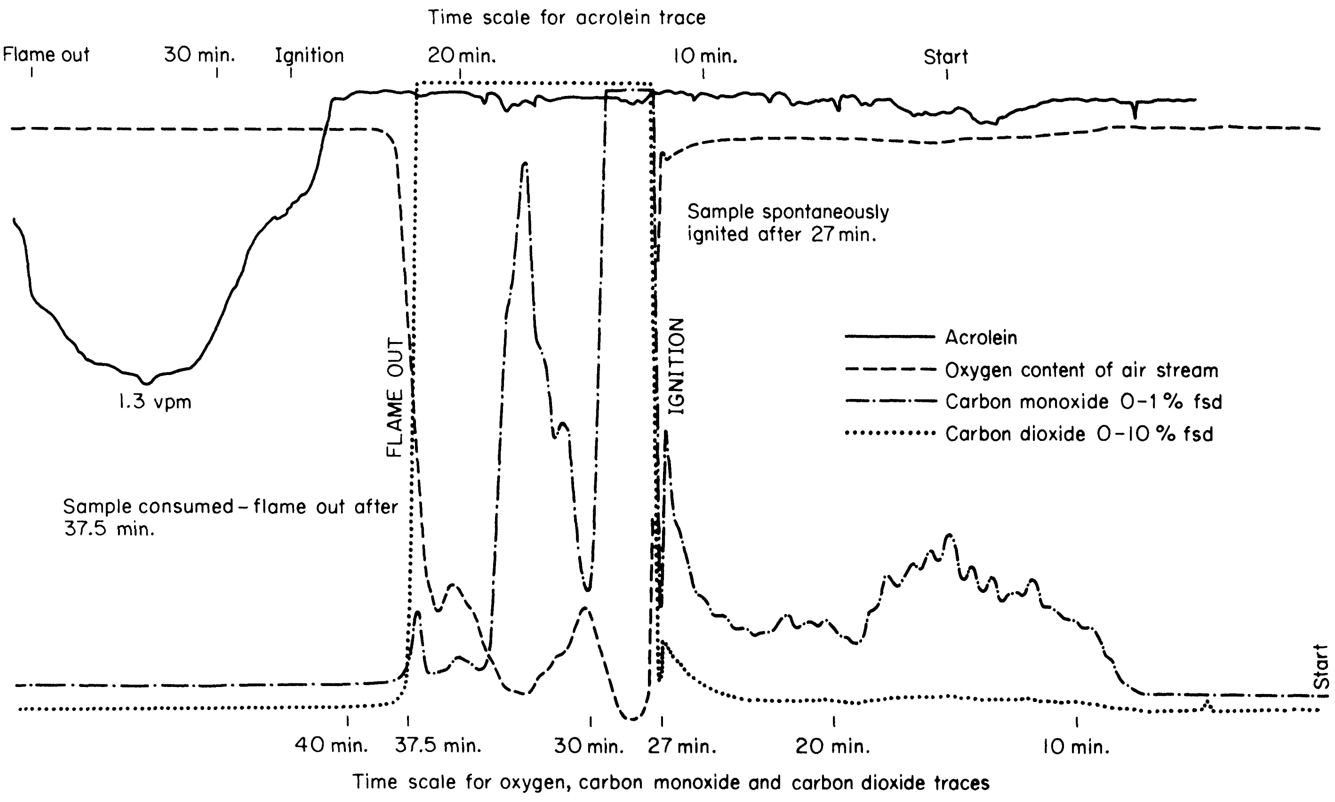

*Figure 4. Pyrolysis at 600°C in air of poly(methyl methacrylate) in a moving tubular furnace (DIN 53436).*

### Applications and discussion

The anlyser has behaved reliably for several investigations: including the measurement of pyrolysis and combustion products from polymethyl methacrylate, pyrolysis products from glass-filled polyethylene terephthalate and pyrolysis/combustion products from polyolefines.

A typical record obtained during the pyrolysis in air of a sample of poly(methylmethacrylate) in a moving tubular furnace (DIN 53436) at 600°C is shown in figure 4. Real-time traces for carbon monoxide, carbon dioxide and oxygen in the exit gas flow from the pyrolyser are also shown. The 14 min. delay due to the reaction coil of the acrolein analyser is clearly evident. Other results obtained confirm that acrolein, together with other fumes, including carbon monoxide and formaldehyde, is evolved during the processing of plastics within the temperature range 140–340°C. The amounts, however, are generally low and even at the highest temperatures the evolved gases are adequately handled by the ventilation installed [3, 4].

### Acknowledgements

I am grateful for assistance of members of the Laboratory Apparatus Section at ICI in the construction of parts of this assembly, and am indebted to the directors of ICI (Petrochemicals and Plastics Division) for permission to publish this paper.

### References

1. Kuwata, K., Ulbori, M., and Yoshiaki, J., *Chromatographic Science*, **17** (1979), 264.

2. Cohen, I. R., and Altshuller, A. P., *Analytical Chemistry*, **33** (1961), 726.

3. Edgerley, P. G., *Fire & Materials*, **4** (1980), 77–82.

4. Edgerley, P. G., *Plastic & Rubber Processing and Applications*, **1** (1981), 81–86.

